# miR-370 and miR-373 regulate the pathogenesis of osteoarthritis by modulating one-carbon metabolism via SHMT-2 and MECP-2, respectively

**DOI:** 10.1111/acel.12363

**Published:** 2015-06-24

**Authors:** Jinsoo Song, Dongkyun Kim, Churl-Hong Chun, Eun-Jung Jin

**Affiliations:** 1 Department of Biological Sciences, College of Natural Sciences, Wonkwang UniversityIksan, Chunbuk, 570-749, Korea; 2 Department of Orthopedic Surgery, Wonkwang University School of MedicineIksan, Chunbuk, 570-749, Korea

**Keywords:** human articular chondrocyte, miR-370/373, one-carbon metabolism, osteoarthritis, SHMT-2/MECP-2, total knee replacement

## Abstract

The aim of this study was to determine the mechanism underlying the association between one-carbon metabolism and DNA methylation during chronic degenerative joint disorder, osteoarthritis (OA). Articular chondrocytes were isolated from human OA cartilage and normal cartilage biopsied, and the degree of cartilage degradation was determined by safranin O staining. We found that the expression levels of SHMT-2 and MECP-2 were increased in OA chondrocytes, and 3′UTR reporter assays showed that SHMT-2 and MECP-2 are the direct targets of miR-370 and miR-373, respectively, in human articular chondrocytes. Our experiments showed that miR-370 and miR-373 levels were significantly lower in OA chondrocytes compared to normal chondrocytes. Overexpression of miR-370 or miR-373, or knockdown of SHMT-2 or MECP-2 reduced both MMP-13 expression and apoptotic cell death in cultured OA chondrocytes. *In vivo*, we found that introduction of miR-370 or miR-373 into the cartilage of mice that had undergone destabilization of the medial meniscus (DMM) surgery significantly reduced the cartilage destruction in this model, whereas introduction of SHMT-2 or MECP-2 increased the severity of cartilage destruction. Together, these results show that miR-370 and miR-373 contribute to the pathogenesis of OA and act as negative regulators of SHMT-2 and MECP-2, respectively.

## Introduction

Osteoarthritis (OA) was previously considered a degenerative disease but is now believed to be a metabolically dynamic process; it takes a worldwide toll in terms of decreased physical ability, increased morbidity, and a high utilization of healthcare resources (Corti & Rigon, 2003). With the exception of total knee replacement (TKR) surgery, there is currently no safe, long-term, and effective treatment for OA pain (Wenham *et al*., 2013). Methotrexate (MTX) is probably the most widely used antisynovial treatment for inflammatory arthritis (Yazici, 2010). MTX treatment of chondrocytes has been shown to increase the uptake of [^3^H]-thymidine while decreasing the uptake of [^3^H]-d-uridine, which reflects altered DNA metabolism (Neidel *et al*., 1998). This suggests that the maintenance or repair of the thymidylate biosynthetic pathway in OA-afflicted chondrocytes may be an effective therapeutic target.

The term ‘one-carbon metabolism’ refers to a system of interdependent metabolic pathways that facilitate the transfer of the one-carbon units that are needed for DNA methylation, dTMP synthesis, and purine synthesis (Stover, 2009). It also produces several metabolic intermediates that are used in the homocysteine and folate metabolic pathways. Methylation is responsible for controlling gene expression, stabilizing the chromatin structure, and maintaining genomic stability; these DNA-methylation-mediated epigenomic changes may regulate the expression of pathogenic genes involved in various diseases (Nishida & Goel, 2011). However, the functional roles and regulatory mechanisms that connect one-carbon metabolism, methylation, and OA have not yet been studied in detail.

During the past two decades, numerous studies have identified and emphasized the importance of microRNAs (miRNAs), which are endogenous small noncoding RNAs that regulate gene expression by pairing with a complementary site in the 3′UTR to interfere with the translation or stability of target transcripts (Jackson & Linsley, 2010). Our understanding of miRNA biogenesis has increased, and numerous studies have shown that miRNAs play important roles in the pathogenesis of various diseases (Brodeur, 2003; Tonge *et al*., 2013). In an effort to define the molecular mechanisms underlying OA, researchers are currently examining the roles of specific miRNAs in the morphological transition, apoptosis, and/or regulation of chondrocyte-specific genes (Miyaki *et al*., 2010). For example, the major miRNA, miR-140, has been shown to function in chondrogenesis and cartilage development. Deletion of miR-140 predisposed mice to develop age-related OA-like changes and further increased cartilage destruction in an OA animal model by directly targeting A disintegrin and metalloproteinase with thrombospondin motifs 5 (ADAMTS-5), aspartyl aminopeptidase (Dnpep), and insulin-like growth factor binding protein 5 (IGFBP-5). More recently, miR-140 was shown to directly mediate matrix metalloprotease (MMP)-13 expression *in vitro* (Tardif *et al*., 2009). Recent studies have also revealed that several other miRNAs are involved in the pathogenesis of OA. For example, miR-9 contributes to regulating MMP-13, miR-98 and miR-146, which are important for controlling the expression of tumor necrosis factor α (Jones *et al*., 2009). Various epi-miRNAs, including miR-29, miR-152, and miR-290, have been shown to play pivotal roles in regulating the epigenetic modifications that occur through DNA methylation (Ji *et al*., 2013). miR-29a (Benetti *et al*., 2008), miR-152 (Fabbri *et al*., 2007), and the miR-17∼29 cluster (Dakhlallah *et al*., 2013) have all been shown to target DNA methyltransferase (DNMT), a key regulator of DNA methylation, thereby contributing to hepatocellular carcinoma, pulmonary fibrosis, and 16HBE cells. In terms of cartilage, miR-140 targets histone deacetylase (HDAC) 4, which controls chondrocyte hypertrophy during skeletogenesis (Tuddenham *et al*., 2006) and controls cartilage homeostasis by regulating ADAMTS-5 and MMP-13 to protect against cartilage damage (Miyaki & Asahara, 2012). Growing evidence from cartilage-based experiments suggests that the epi-miRNAs involved in regulating various pathogenesis-related genes may be useful targets for the diagnosis, prevention, and treatment of OA. However, we need a detailed understanding of the regulatory network governing these epi-mRNAs if we hope to use them in therapeutic applications for controlling OA.

There is currently no disease-modifying therapy available for patients with OA (Jordan *et al*., 2003). Growing evidence suggests that unraveling the role of miRNAs in joint physiology and pathology may facilitate the diagnosis, prevention, and treatment of OA. However, we must understand the network of expressed mRNAs and their relationships with cognate genes to further develop miRNAs for the therapeutic control of OA. In this prospective study, we examined the potential functional role of miR-370 and miR-373 in OA pathogenesis. We identify serine hydroxymethyltransferase (SHMT)-2 and methyl-CpG-binding protein (MECP)-2 as targets of miR-370 and miR-373, respectively, and hypothesize that these interactions contribute to the pathogenesis of OA. Finally, we propose that miR-370 and miR-373 could be potent therapeutic targets for OA.

## Result

**Fig 1:**
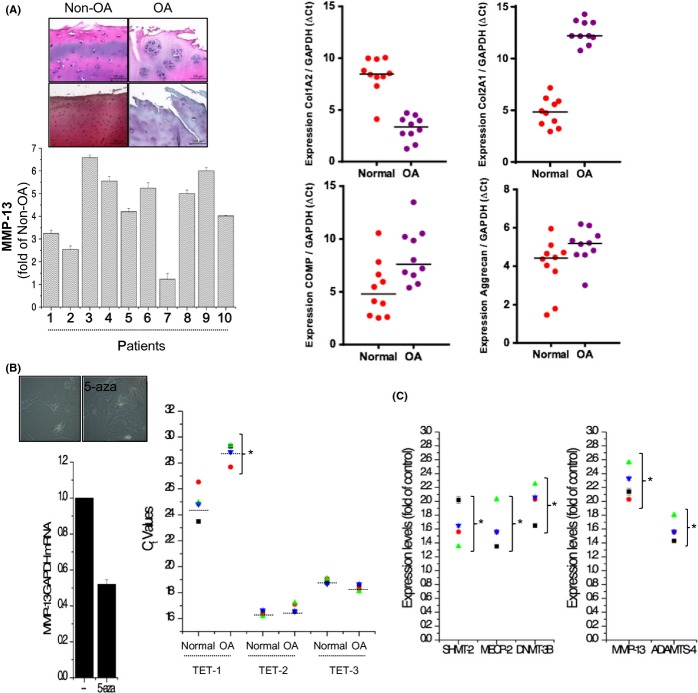
The involvement of methylation during OA pathogenesis. (A) OA cartilages (*n* = 10) were divided into healthy zones (Non-OA) and severely damaged zones (OA) and stained with safranin O (left panel). Normal chondrocytes were isolated form biopsy of normal cartilage, OA chondrocytes were isolated from OA area from the 10 patient samples, and the expression levels of type I and type II collagen, aggrecan, and COMP were analyzed by real-time PCR (right panel). (B) OA chondrocytes were treated with the methyltransferase inhibitor, 5-azacytidine (5-aza). Cell images were captured, and MMP-13 expression was analyzed by real-time PCR (left panel). The expression levels of TET-1, TET-2, and TET-3 were analyzed in chondrocytes isolated from normal biopsy samples (Normal) and OA chondrocytes. (C) Normal and OA chondrocytes (*n* = 4 samples in each case) were subjected to real-time PCR analysis of SHMT-2, MECP-2, DNMT-3B, MMP-13, and ADAMTS-4 expression. *P < 0.05 vs. control (normal). *, statistically different from normal (*P* < 0.05).

Cartilage samples were obtained from patients with OA (*n* = 10; average age, 72.6) undergoing total knee replacement surgery; they were divided into non-OA (a relatively heathy area of OA cartilage, non-OA chondrocytes) and OA regions (a severely damaged area of OA cartilage, OA chondrocytes), and articular chondrocytes were isolated and cultured. Normal chondrocytes were isolated from biopsy samples of normal cartilage (up to 0.1 g) (*n* = 10; average age, 45.6). Consistent with a previous report (Song et al., 2013), we observed loss of the cartilage matrix as analyzed by safranin O staining with drastic severe degradation in damaged area of OA cartilage (OA) compared to relatively healthy area of OA cartilage (non-OA) and increased MMP-13 levels in OA chondrocytes compared to non-OA chondrocytes (Fig. 1A). Furthermore, type I collagen, type II collagen, aggrecan, and COMP levels were analyzed to confirm the intrinsic characteristics of normal or OA chondrocytes (Fig.1A). Epigenetic mechanisms (e.g., DNA methylation, chromatin modification, and noncoding RNAs) have been suggested to regulate the OA-specific genes responsible for cartilage degradation (Hashimoto *et al*., 2009). Here, we found that treatment with the DNA methyltransferase inhibitor, 5-azacytidine, reduced the level of MMP-13 (Fig.1B). DNA methylation at the 5 position of cytosine (5-mC), which can be converted to 5-hydroxymethylcytosine (5-hmC) by the ten-eleven translocation (TET) family proteins, has emerged as a key epigenetic marker that plays essential roles in various biological and pathological processes (Ito *et al*., 2011). We also found that the expression level of TET-1 was significantly lower in OA chondrocytes compared to normal chondrocytes which were isolated from biopsy sample of normal cartilages (*n* = 5; average age, 45.3) (Fig.1B). These data suggest that dysregulation of methylation may be responsible for OA-inducing events.

Folate status can affect the degree of global DNA methylation and has been associated with gene silencing, indicating that epigenetic gene silencing during development might be modified by one-carbon metabolism (Gonzalez, 2013). Furthermore, a recent study suggested that one-carbon metabolism may be involved in OA pathogenesis, and proposed that regulation of this pathway could be a therapeutic target for OA. Here, we found that exposure of OA chondrocytes to folate increased the levels of SHMT-2, MECP-2, DNMT-3B (all of which are involved in the methylation process), MMP-13, and ADAM-TS (Fig.1C).

**Fig 2:**
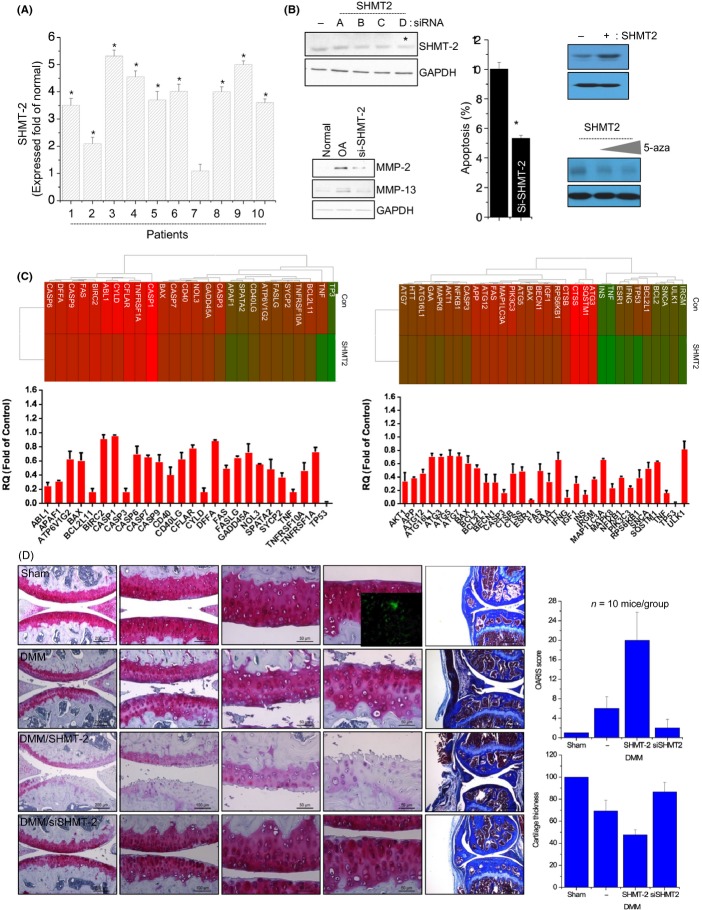
SHMT-2 is involved in OA pathogenesis *in vitro*. (A) OA chondrocytes (*n* = 10 patients) were subjected to real-time PCR analysis of SHMT-2 expression and represented as fold of normal chondrocytes. (B) SiRNA-mediated knockdown of SHMT-2 was confirmed by immunoblotting (left upper panel), and the protein levels of MMP-2 and MMP-13 (left lower panel) were analyzed by immunoblotting and apoptosis (left right) was analyzed by Muse™ Cell Cycle assay. (C) Gene profile of apoptosis-related genes (left panel) and autophagy-related genes (right panel) was examined and expressed as heat map (upper panel) and bar graph (lower panel). ‘Red’ color represents significant decrease in expression levels. Bars show the mean ± SD of three individual experiments. (D) Mice were subjected to destabilization of the medial meniscus (DMM) surgery as a model for OA and infected with lentiviruses expressing SHMT-2 (SHMT2) or its specific siRNA (siSHMT-2). DMM and sham-operated (Sham, control) cartilages were stained with safranin O. Cartilage destruction was scored according to the guidelines of the OARSI histopathology initiative, and the average thickness of cartilage was plotted. Inserted image showed the successful lentiviral infection. Mouse cartilage was scrapped into slide and captured image under fluorescent microscopy. *P < 0.05 vs. control (normal). *, statistically different from normal (*P* < 0.05).

The exact regulatory mechanisms that modulate one-carbon metabolism have not been well studied, although SHMT-1, SHMT-2α, thymidylate synthase (TYMS), and dihydrofolate reductase (DHFR) are known to be involved (Anderson & Stover, 2009). Methylene-THF, which is generated by SHMT, acts as a one-carbon donor for the TYMS-catalyzed conversion of dUMP to thymidylate, generating dihydrofolate. Thereafter, DHFR catalyzes the NADPH-dependent reduction of dihydrofolate to regenerate THF for subsequent cycles of *de novo* thymidylate synthesis (Anderson *et al*., 2012). Therefore, we examined the expression level of SHMT-2 in OA and normal chondrocytes. The expression level of SHMT-2 was increased in OA chondrocytes compared to normal chondrocytes (Fig.2A). To further investigate the role of SHMT-2, we introduced four different small interfering RNAs (siRNAs) against SHMT-2 into the chondrocyte cell line. The knockdown of SHMT-2 by all four siRNAs, which was confirmed by immunoblotting (Fig.2B, left upper panel), was found to reduce protein levels of MMP-2 and MMP-13 in OA chondrocytes compared to normal chondrocytes (Fig.2B, left lower panel). Chondrocyte apoptosis was significantly increased with knockdown of SHMT-2 (Fig.2B, middle panel). On the other hand, overexpression of SHMT-2 significantly induced MMP-13 protein level and this increased MMP-13 protein was suppressed by cotreatment of 5-azacytidine (Fig.2B, right panel) providing the insight that SHMT-2 mediates MMP-13 methylation during OA pathogenesis.

Moreover, RNA levels of apoptosis-related genes and autophagy-related genes were significantly downregulated by knockdown of SHMT-2 in OA chondrocytes (Fig.2C). In *in vivo* DMM OA model, cartilage destruction as visualized by safranin O and trichrome staining and OARSI scoring was significantly increased by overexpression of SHMT-2, whereas knockdown of SHMT-2 successfully blocked cartilage degradation induced by DMM surgery (Fig.2D).

**Fig 3:**
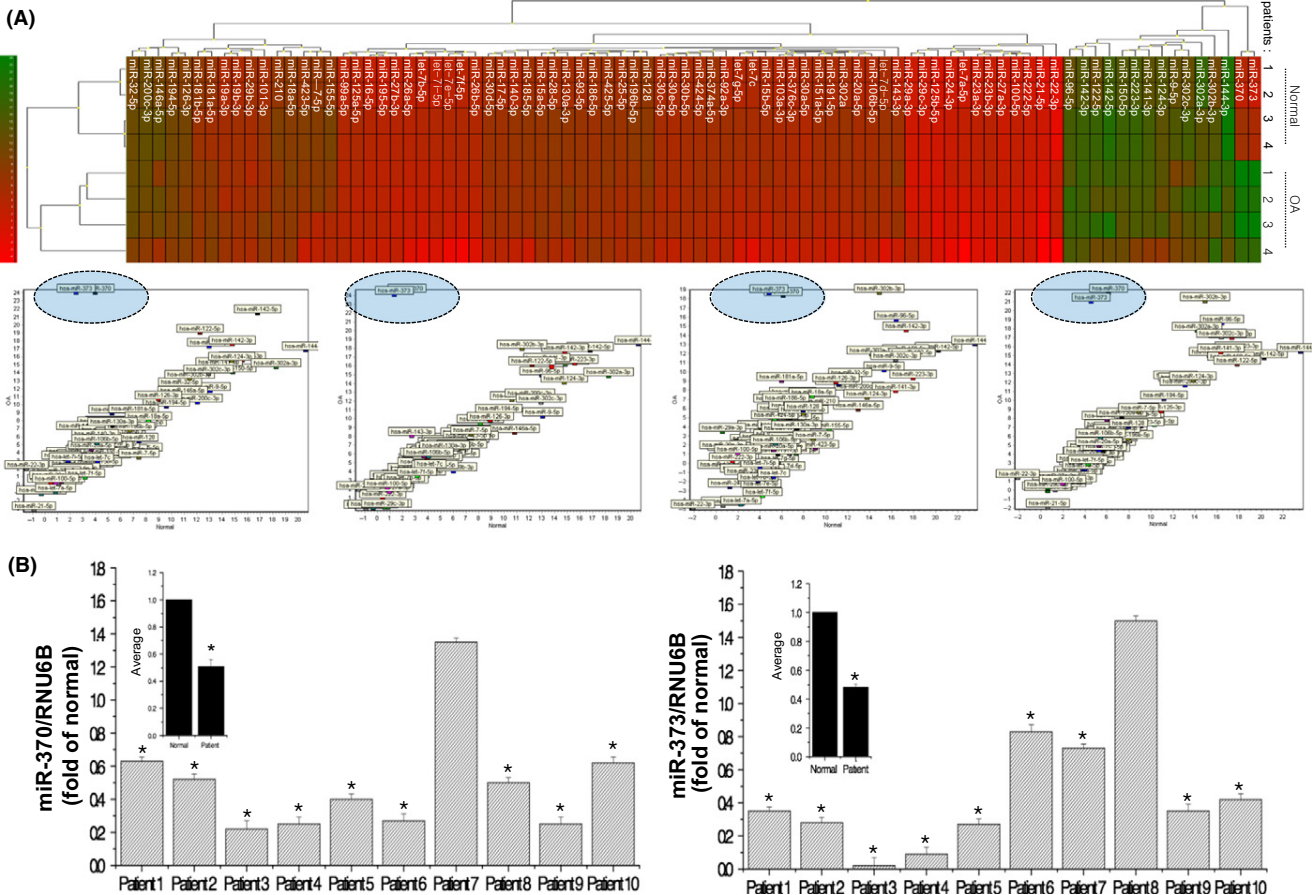
miR-370 and miR-373 are involved in OA pathogenesis. (A) Expression levels of miRNAs were analyzed using normal chondrocytes (*n* = 4) isolated from biopsy sample of normal cartilage and OA chondrocytes (*n* = 4) isolated from severely damaged OA cartilage and presented as heat map. ‘Red’ color represents significant decrease in expression levels (upper panel). Examples of correlation graph on miRNA profile between normal vs. OA chondrocytes are shown (lower panel). (B) Expression levels of miR-370 and miR-373 were analyzed in OA chondrocytes (*n* = 10) isolated from severely damaged OA cartilage, compared to average value of normal chondrocytes (*n* = 4), and represented as fold of normal chondrocytes. *P < 0.05 vs. control (normal). *, statistically different from normal (*P* < 0.05).

**Fig 4:**
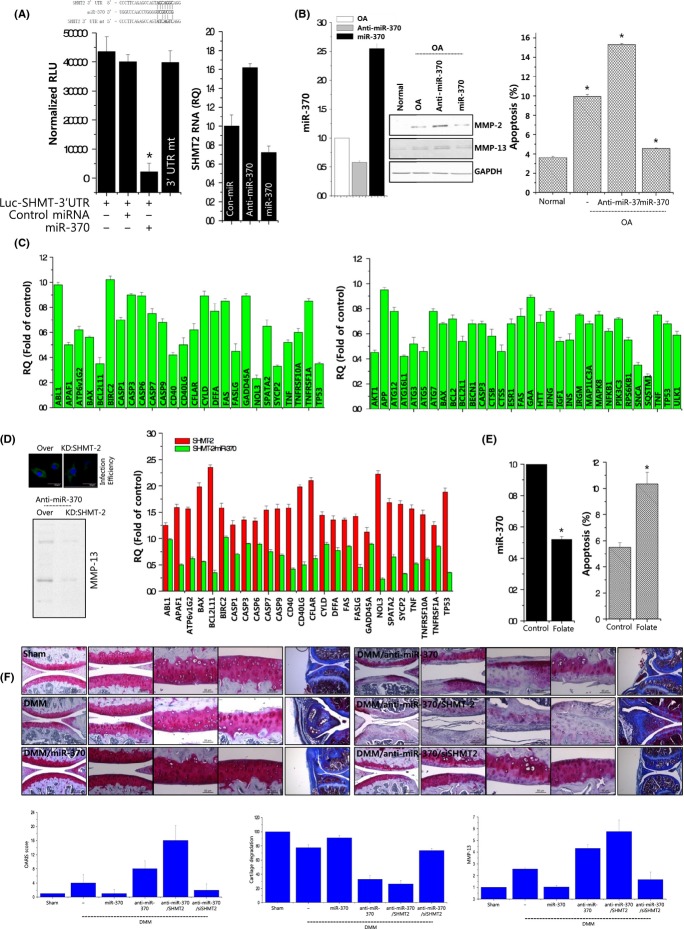
miR-370 regulates OA pathogenesis by targeting SHMT-2. (A) Luciferase reporter activity was driven by the 3′UTR of SHMT-2 or mutated 3′UTR of SHMT-2 (3′UTR mt) with or without forced expression of miR-370 (left panel). OA chondrocytes were treated with miR-370 precursor (miR-370) and miR-370 inhibitor (anti-miR-370), and SHMT-2 level was analyzed by real-time PCR (right panel). (B) The efficiencies of the miR-370 precursor (miR-370) and miR-370 inhibitor (anti-miR-370) in normal chondrocytes were analyzed by real-time PCR (left panel). Normal and OA chondrocytes were treated with a miR-370 precursor (miR-370) or an inhibitor of miR-370 (anti-miR-370), and the protein expression levels of MMP-2 and MMP-13 were analyzed by immunoblotting. GAPDH was used as a loading control (middle panel). Apoptosis was analyzed by Muse™ Cell Cycle assay (right panel). (C) Gene profile of apoptosis-related genes (left panel) and autophagy-related genes (right panel) was examined and expressed as bar graph. Bars show the mean ± SD of three individual experiments. (D) OA chondrocytes were infected with SHMT-2-expressing lentiviruses or SHMT-2-specific siRNAs in the presence of anti-miR-370. The infection efficiency was assessed by fluorescent microscopy as both SHMT-2-expressing lentiviruses and SHMT-2-specific siRNAs were tagged with GFP, and MMP-13 protein expression was analyzed by immunoblotting (left panel). Gene profile of apoptosis-related genes was examined and expressed as bar graph. Bars show the mean ± SD of three individual experiments (right panel). (E) OA chondrocytes were treated with folate, and miR-370 expression was analyzed by real-time PCR (left panel) and apoptosis was analyzed by Muse™ Cell Cycle analysis (right panel). (F) DMM and sham-operated (Sham) mice were infected with miR-370 precursor (miR-370)- or the miR-370 inhibitor (anti-miR-370)-encoding lentiviruses in the presence or absence of SHMT2 (SHMT-2)- or SHMT-2-specific siRNA (siSHMT-2)-encoding lentiviruses, and cartilage samples were stained with safranin O. Cartilage destruction was scored and average cartilage thickness was plotted. RNA was isolated from cartilage sections, and the expression level of MMP-13 was analyzed by real-time PCR. *P < 0.05 vs. control (normal). *, statistically different from normal (*P* < 0.05).

In an effort to identify the regulatory molecules that act on SHMT-2, we focused on miRNAs, which affect many biological and pathological responses by targeting key functional genes. We analyzed the expression patterns of miRNAs using normal chondrocytes (*n* = 4) isolated from biopsy sample of normal cartilage and OA chondrocytes (*n* = 4) isolated from OA cartilage. We searched miRNAs that involved in Epigenetic Regulation Database as well as degenerative diseases such as OA using several miR databases (www.mirbase.org, www.mirdb.org, www.targetscan.org, www.microrna.org, http://210.46.85.180:8080/EpimiR) and selected miRNAs to analyze. Among we analyzed, miR-370 and miR-373 seem highly OA specific (Fig.3A). Furthermore, decreased levels of miR-370 and miR-373 were observed in OA chondrocytes from majority of patients, as confirmed by analysis of MMP-13 expression (Fig.3B). To see whether these two OA-specific miRNAs are involved in the regulation of SHMT-2, we cloned the entire 3′UTR of SHMT-2 into a luciferase reporter vector, electroporated the vector into cells along with the precursor of miR-370 or a cognate nontargeting negative control, and assayed cell lysates for luciferase expression. We found that chondrocytes transfected with the SHMT-2 3′UTR-driven vector plus pre-miR-370 exhibited significantly less luciferase activity but mutated SHMT-2 3′UTR-driven vector plus pre-miR-370 did not alter luciferase activity (Fig.4A, left panel) compared to cells that received the reporter plus the nontargeting negative control, suggesting that miR-370 may be involved in the SHMT-2-regulated pathogenesis of OA. However, chondrocytes transfected with the SHMT-2 3′UTR-driven vector plus pre-miR-373 showed no difference in luciferase activity compared to cells that received the reporter plus the nontargeting negative control (data not shown). In addition, induction of miR-370 decreased SHMT-2 RNA level, whereas knockdown of miR-370 increased SHMT-2 RNA level in OA chondrocytes (Fig.4A, right panel).

To investigate whether modulation of miR-370 induced the same effects as modulation of SHMT-2, we altered the expression level of miR-370 using its specific inhibitor or precursors (Fig.4B). Introduction of miR-370 as confirmed by real-time PCR into OA chondrocytes reduced the protein levels of MMP-2 and MMP-13 that are typically increased in OA chondrocytes. On the other hand, inhibition of miR-370 in OA chondrocytes increased the expression levels of these proteins. In addition, annexin V staining revealed that apoptotic cell death was increased in OA chondrocytes (9.94%) compared to normal chondrocytes (3.62%). However, the induction of miR-370 in OA chondrocytes significantly reduced this apoptotic cell death to 4.56%, whereas inhibition of miR-370 increased cell death among OA chondrocytes. Overexpression of miR-370 suppressed the RNA levels of apoptosis-related and autophagy-related genes (Fig.4C). Co-induction of miR-370 significantly inhibited SHMT-2-induced MMP-13 expression and SHMT-2-upregulated apoptosis-related gene cell death in normal chondrocytes (Fig.4D). In addition, folate treatment reduced miR-370 levels and apoptosis of OA chondrocytes (Fig.4E), suggesting that miR-370 may be involved in one-carbon metabolism. In *in vivo* study using DMM mice, the most severe cartilage destruction (as visualized by safranin O staining) was observed among mice infected with anti-miR-370- and SHMT-2-encoding lentiviruses, whereas infection of DMM mice with miR-370-expressing lentiviruses significantly ameliorated DMM-induced cartilage destruction (Fig.4F). The MMP-13 RNA level as examined using RNA extracted from paraffin sections showed same expression pattern with the degree of cartilage degradation.

**Fig 5:**
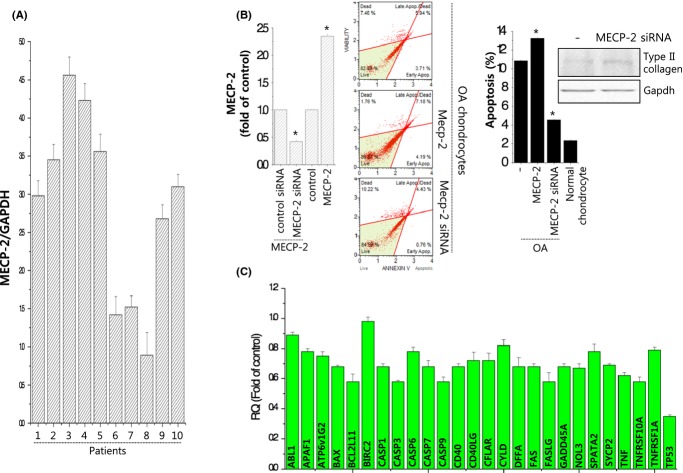
MECP-2 is involved in OA pathogenesis *in vitro*. (A) RNA level of MECP-2 was analyzed using OA chondrocytes (*n* = 10), compared to average value of normal chondrocytes (*n* = 4), and represented as fold of normal. (B) Chondrocytes were infected with MECP-2- or MECP-2 siRNA-encoding lentiviruses. Efficiency was confirmed by real-time PCR (left panel), viability was analyzed by annexin V staining (middle panel), apoptosis was analyzed with the Muse™ Cell Cycle assay (right panel), and the protein level of type II collagen was analyzed by immunoblotting (right upper panel). GAPDH was used as a loading control. (C) Gene profile of apoptosis-related genes (left panel) and autophagy-related genes (right panel) was examined with knockdown of MECP-2 in OA chondrocytes. *P < 0.05 vs. control (normal). *, statistically different from normal (*P* < 0.05).

Studies have shown that 5-mC can inhibit gene transcription by attracting methyl-CpG-binding domain (MBD)-containing proteins (e.g., methyl-CpG-binding protein; MECP-2); these proteins ‘read’ methylation marks, bind to methylated DNA at methylation sites (Srinivasan *et al*., 2013), and affect chromatin condensation by recruiting corepressor proteins (e.g., SIN3A and histone-modifying enzymes) (Kadonaga, 1998). We found that the expression level of MECP-2 was significantly increased in OA chondrocytes compared to normal chondrocytes (Fig.5A). To further examine the potential role of MECP-2 in OA pathogenesis, we modulated MECP-2 level exogenously using its specific siRNA or overexpression vector and confirmed the efficiency of MECP-2-specific siRNA or MECP overexpression vector using real-time PCR (Fig.5B). Overexpression of MECP-2 increased apoptosis as assessed by annexin V staining in OA cell chondrocytes, and knockdown of MECP-2 reduced apoptosis and induced the protein level of type II collagen in OA chondrocytes. In addition, knockdown of MECP-2 significantly inhibited apoptosis-related genes in OA chondrocytes (Fig.5C).

**Fig 6:**
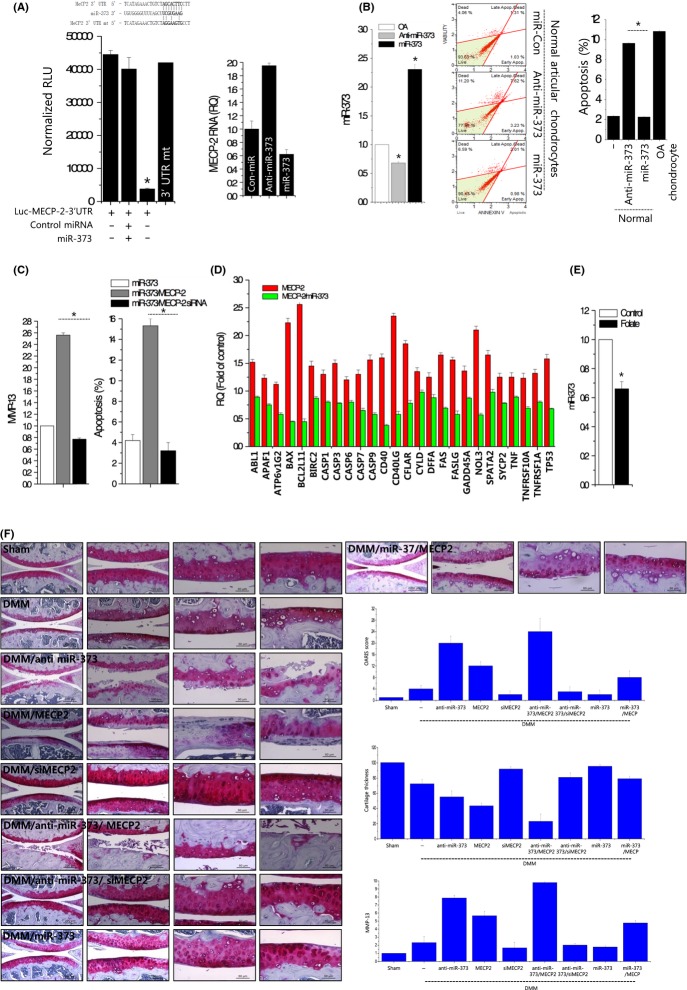
miR-373 regulates OA pathogenesis by targeting MECP-2. (A) Luciferase reporter assays were performed on OA chondrocytes harboring a vector construct driven by the 3′UTR of MECP-2 or mutated 3′UTR of MECP-2 (3′UTR mt) with or without forced expression of miR-373 (left panel). RNA level of MECP was examined with treatment of the miR-373 precursor (miR-373) or miR-373 inhibitor (anti-miR-373) (right panel). (B) Chondrocytes were treated with miR-373 precursor (miR-373) or miR-373 inhibitor (anti-miR-373), the efficiency was confirmed by real-time PCR (left panel), viability was analyzed by annexin V staining (middle panel), and apoptosis was analyzed with the Muse™ Cell Cycle assay (right panel). (C) Normal chondrocytes were treated with MECP-2-expressing vector or the MECP-2-specific siRNA (MECP-2 siRNA) in the presence of the miR-373 precursor (miR-373). RNA level of MMP-13 was analyzed by real-time PCR, and apoptosis was analyzed by Muse™ Cell Cycle assay. (D) Gene profile of apoptosis-related genes was examined and expressed as bar graph. Bars show the mean ± SD of three individual experiments. (E) OA chondrocytes were treated with folate, and miR-373 expression was analyzed by real-time PCR. (F) DMM and sham-operated mice were infected with lentiviruses encoding the miR-370 precursor (miR-373)/miR-370 inhibitor (anti-miR-370) or MECP-2/MECP-2-specific siRNA (siMECP2), or combination of miR-370 precursor (miR-373)/miR-370 inhibitor (anti-miR-370) and MECP-2/MECP-2-specific siRNA (siMECP2), and cartilage samples were stained with safranin O. Cartilage destruction was scored according to the guidelines of the OARSI histopathology initiative, and the average thickness of cartilage was plotted. RNA was isolated from cartilage sections, and the expression level of MMP-13 was analyzed by real-time PCR. *P < 0.05 vs. control (normal). *, statistically different from normal (*P* < 0.05).

To further test whether MECP-2 is a target of miR-370 or miR-373, we cloned the entire 3′UTR of MECP-2 into a luciferase reporter vector, electroporated the vector into chondrocytes along with the precursor of miR-370 or miR-373 or a cognate nontargeting negative control, and assayed cell lysates for luciferase expression. We found that cells transfected with the MECP-2 3′UTR-driven vector plus pre-miR-373 exhibited significantly less luciferase activity, but mutated MECP-2 3′UTR-driven vector plus pre-miR-373 did not alter luciferase activity compared to cells that received the reporter plus the nontargeting negative control (Fig.6A, left panel). In addition, induction of miR-373 decreased RNA level of MECP-2, whereas knockdown of miR-373 increased RNA level of MECP-2 in OA chondrocytes (Fig.6A, right panel). Next, to investigate whether modulation of miR-373 induced the same effects as modulation of MECP-2, we altered the expression level of miR-373 using its specific inhibitor or precursors (Fig.6B). Annexin V staining revealed that the induction of miR-373 in OA chondrocytes significantly reduced this apoptotic cell death to 2.26%, whereas inhibition of miR-370 increased cell death up to 10% in normal chondrocytes. Overexpression of MECP-2 significantly increased MMP-13 mRNA expression and apoptosis, whereas siRNA-mediated knockdown of MECP-2 significantly decreased MMP-13 mRNA expression and apoptosis (Fig.6C). Co-induction of miR-373 significantly inhibited MECP-2-upregulated apoptosis-related gene cell death in normal chondrocytes (Fig.6D). In addition, folate treatment decreased the level of miR-373 in OA chondrocytes suggesting that miR-373 may be involved in one-carbon metabolism (Fig.6E). In *in vivo* study using DMM mice, overexpression of MECP2 and knockdown of miR-373 induced severe cartilage degradation, whereas knockdown of MECP2 and overexpression of miR-373 successfully blocked cartilage degradation induced by DMM surgery. The most severe cartilage destruction (as visualized by safranin O staining and OARSI scoring) was observed among mice infected with the combination of anti-miR-373- and MECP2-encoding lentiviruses (Fig.6F). The MMP-13 RNA level as examined using RNA extracted from paraffin sections showed same expression pattern with the degree of cartilage degradation.

## Discussion

One-carbon metabolism is linked to DNA synthesis, DNA methylation, amino acid metabolism, and cell proliferation, and dysfunctions of genes involved in one-carbon metabolism have been associated with increased risks for various diseases, including cancer and anemia (Jones *et al*., 1998; Coppedè, 2014). DNA methylation, which is a crucial epigenetic alteration, is subject to particular control by one-carbon metabolism. SHMT is an important protein in one-carbon metabolism; it catalyzes the reversible conversion of glycine to serine, which provides metabolites (e.g., formyl groups) that are important for purine biosynthesis, and the methyl groups needed for pyrimidine synthesis, homocysteine remethylation, and other reactions that are important for cellular homeostasis (Locasale, 2013). The folate metabolites generated via the action of SHMT play important role in maintaining normal methylation patterns, DNA stability, and genetic variation (Blom & Smulders, 2011). SHMT-1 and SHMT-2 have been associated with a wide variety of human phenotypes, including neural tube defects (Duthie, 2011), childhood acute leukemia (Relton *et al*., 2004), rectal carcinoma (Vijayakrishnan & Houlston, 2010), and prostate cancer (Komlósi *et al*., 2010). Shmt1+/− mice accumulate uracil within nuclear DNA (Collin *et al*., 2009) and show increased susceptibility to neural tube defects (Collin *et al*., 2009) and intestinal cancer (MacFarlane *et al*., 2008). Another important set of proteins involved in one-carbon metabolism are the DNA methyltransferases (DNMTs). DNMT dysfunction has been associated with impaired memory consolidation, decreased synaptic plasticity, memory deficits, and learning disabilities (Beaudin *et al*., 2011). DNMT-3B gene mutations have been shown to cause the immunodeficiency–centromeric instability–facial anomalies syndrome, which is characterized by hypomethylation of pericentromeric repeats (Macfarlane *et al*., 2011). DNMT is recruited by MECP-2, which acts as a transcriptional regulator by modulating the expression of methylation-sensitive genes. Notably, MECP-2 deficiency has been associated with Rett syndrome, a neurological disorder in humans. In the present study, we show that SHMT-2 and MECP-2 are upregulated in OA chondrocytes, and overexpression of both SHMT-2 and MECP-2 induced severe cartilage degradation in the DMM mouse model. This supports the idea that misregulation of one-carbon metabolism may be involved in the pathogenesis of OA.

Recently, Stone and colleagues performed a Monte Carlo-based *in silico* analysis and found that several miRNAs could play important roles in regulating one-carbon metabolism (Monsey *et al*., 2011). They suggested miR-22 and miR-125 as possible master regulators, and miR-344-5p/484 and miR-488 as possible master coregulators that may influence the genes involved in one-carbon metabolism. However, we do not yet fully understand the detailed functions of miRNAs in modulating one-carbon metabolism. Here, we present the first evidence suggesting that miR-370 and miR-373 may potently regulate one-carbon metabolism by directly targeting SHMT-2 and MECP-2, respectively.

Studies comparing the expression levels of miRNAs between OA tissue specimens and normal cartilage tissue/non-OA tissue specimens have identified several miRNAs as being involved in the pathogenesis of OA. For example, Iliopoulos *et al*. showed that 16 miRNAs (nine upregulated and seven downregulated) were differentially expressed in OA cartilage compared with normal controls. Seventeen miRNAs whose expression varied by fourfold or more in normal vs. late-stage OA cartilage were identified. Of these differentially expressed miRNAs, miR-27b (downregulated in OA) directly targets MMP-13 expression (Stone *et al*., 2011); miR-22 (upregulated in OA) directly regulates PPARA and BMP-7 expression in cartilage; miR-9 inhibits MMP13 secretion in isolated human chondrocytes; and miR-146a is highly expressed in early OA cartilage and has been shown to control knee joint homeostasis and OA-associated algesia by balancing inflammatory responses in the cartilage and synovium. Here, we report the first study showing that the levels of miR-370 and miR-373 are significantly lower in OA chondrocytes. The previously identified targets for miR-370 and miR-373 were largely associated with cancer pathogenesis (e.g., oncogenes and tumor suppressors), suggesting that the utilized target genes may contribute to the tissue- and cell-type-specific performances of these miRNAs (Akhtar *et al*., 2010). For example, miR-370 targets FOXO1 in gastric mucosal cancer and has been associated with disease progression (Voorhoeve *et al*., 2007). miR-373 regulates MMP activity by modulating MAPK signaling or targeting mTOR and SIRT1 in human fibrosarcoma cells (Fan *et al*., 2013), and by targeting ZIP4 in pancreatic cancer (Liu & Wilson, 2012). Here, we found that miR-370 and miR-373 were significantly downregulated in OA chondrocytes compared to normal chondrocytes. This dysregulates cell survival by targeting the newly discovered targets, SHMT-2 and MECP-2, respectively; apoptosis was induced by the inhibition of miR-370 or miR-373, and also by the overexpression of SHMT-2 or MECP-2. These results suggest that the decreased functions of miR-370 and miR-373 in OA chondrocytes lead to the disinhibition of SHMT-2 and MECP-2, respectively, thereby promoting apoptotic cell death.

In sum, we herein show for the first time that miR-370 and miR-373 directly target and negatively regulate SHMT-2 and MECP-2, respectively, in human articular chondrocytes, where they contribute to the pathogenesis of OA. These results provide novel insights into OA and may facilitate the development of therapeutic approaches based on the modulation of miR-370 and/or miR-373.

## Experimental procedures

### Primary cell cultures

Human articular cartilage specimens were obtained from patients undergoing total knee arthroplasty designated as OA chondrocytes, and normal chondrocytes were obtained from biopsy sample of normal cartilage designated as normal chondrocytes in this study. Tissue collection was approved by the Human Subjects Committee of Wonkwang University (Chunbuk, Korea). Chondrocytes were extracted and seeded at 1.5 × 10^4^ cells cm^−2^ in DMEM (Gibco Invitrogen, Grand Island, NY, USA) supplemented with 10% fetal bovine serum (FBS), 100 units mL^−1^ penicillin, and 100 μg mL^−1^ streptomycin (Gibco Invitrogen).

### Cell viability assay

Muse™ Annexin V (Millipore, Billerica, MA, USA) assay kit and Muse™ apoptosis kit (Millipore) were used to detect apoptotic cell death.

### Quantification of miRNA and real-time quantitative RT–PCR of mRNA

The expression levels of various miRNAs and mRNAs were quantified using the TaqMan microRNA and gene expression assays, respectively (Applied Biosystems: Foster City, CA, USA), according to the manufacturer’s protocols. miRNA expression was normalized with respect to that of the RNU43 small nuclear RNA (endogenous control). For assessment of mRNA, transcripts were quantified by real-time quantitative polymerase chain reaction (RT–PCR) and normalized with respect to the expression of GAPDH.

### miRNA inhibitor-mediated knockdown and pre-miR-370/373-mediated upregulation of miR-370 and miR-373

The precursors or inhibitors of miR-370 and miR-373 (Ambion, Austin, TX, USA) were electroporated into cells using a square-wave generator (BTX-830; Gentronics, San Diego, CA, USA) with 20 ms, 200 square pulses. Scrambled oligos or miRNAs were used as negative controls.

### Reporter vectors and DNA constructs

The full length of 3′ untranslated regions (UTRs) of MECP-2 and SHMT-2 were PCR-amplified and cloned downstream of the CMV-driven firefly luciferase cassette in the pMIR-Report vector (Ambion). For miRNA target validation, cells were electroporated as described above with 25–50 ng of each firefly luciferase reporter construct, 150–175 ng of empty pcDNA3 vector (Clontech, Mountain View, CA, USA), 200 ng pcDNA3 harboring the Renilla luciferase gene (transfection control), and 30 pmol of pre-miR-370, pre-miR-373, or pre-miR-neg (Ambion). At 24 h post-transfection, the activities of the firefly and Renilla luciferase were assayed using commercially available kits (Promega, San Luis Obispo, CA, USA). The ratios of firefly luciferase activity vs. Renilla luciferase activity are given as normalized relative light units (RLUs).

### Production of lentiviral particles

Human 293FT cells were transfected with lentiviral vectors encoding miR-370, miR-373, SHMT-2, or MECP-2 or the negative control lentivirus (Applied Biological Materials, Canada) using the third-generation packaging mix (Applied Biological Materials, Canada) and Lentifectin (Applied Biological Materials, Canada). The cells were then cultured overnight in Opti-MEM I medium (Gibco Invitrogen). The supernatant was collected and lentiviral particles were concentrated using a Lenti-X Concentrator (Clontech).

### Western blotting

Cell lysates were separated by 10% polyacrylamide gel electrophoresis, transferred to a nitrocellulose membrane (Schleicher and Schuell, Dassel, Germany), and probed with antibodies against type II collagen (Santa Cruz Biotech, Santa Cruz, CA, USA), caspase-7 (Cell Signaling, Danvers, MA, USA), MMP-2 (Abcam, Cambridge, MA, USA), MMP-13 (Biovision, San Francisco, CA, USA), and GAPDH (Santa Cruz Biotech). The blots were developed with a peroxidase-conjugated secondary antibody and visualized using an electrochemiluminescence (ECL) system (Pierce Biotechnology Inc., Rockford, MN, USA).

### Arthritic cartilage, experimental OA, and histology of OA cartilage

Human OA cartilage was sourced from individuals undergoing arthroplasty for OA of the knee joint. The Wonkwang University Hospital Institutional Review Board approved the use of these materials, and all individuals provided written informed consent prior to the operative procedure. The human OA cartilage samples were frozen, sectioned at a thickness of 10 μm, fixed in paraformaldehyde, and stained with Alcian blue.

Experimental OA was induced in 8-week-old male mice by destabilization of the medial meniscus (DMM) surgery using C57BL/6 mice. Sham-operated animals injected with empty lentiviruses (mock transduction) were used as controls. DMM surgery was performed in male mice, and lentiviruses were injected intra-articularly with 1 × 10^9^ plaque-forming units (PFU) of lentiviral vectors encoding miR-370, miR-373, SHMT-2, or MECP-2 every week for 8 weeks. The mice were sacrificed 2 weeks after stopping injection and subjected to histological and biochemical analyses. Cartilage destruction in mice was examined using safranin O staining. Briefly, knee joints were fixed in 4% paraformaldehyde, decalcified in 0.5 m EDTA (pH 7.4) for 14 days at 4 °C, and embedded in paraffin. The paraffin blocks were sectioned at a thickness of 6 μm. The sections were deparaffinized in xylene, hydrated with graded ethanol, and stained with safranin O.

### Extraction of RNA from FFPE (formalin-fixed paraffin-embedded) tissues

RNA was extracted from paraffin-embedded samples using a MasterPure kit (Epicentre Biotechnologies, Madison, WI, USA). Briefly, each FFPE tissue was resuspended in MasterPure Tissue and Cell Lysis solution (final concentration, 0.15 mg mL^−1^ proteinase K) and incubated at 65 °C for 30 min. The MasterPure MPC™ protein precipitation reagent was then added, and nucleic acids were precipitated with isopropanol and pelleted by centrifugation at 10 000 × *g* for 10 min at 4 °C. The supernatant was carefully removed. The pellet was washed twice with 75% ethanol, allowed to dry, and then treated with 30 μL TE buffer containing 40 units of RNase inhibitor.

### Statistical analysis

A two-tailed Student’s *t*-test or one-way ANOVA followed by the Student–Newman–Keuls *post hoc* test was used to determine the significance of the differences between results, with *P* < 0.05 taken as indicating a significant difference.

## Funding

This works was supported by National Research Foundation (NRF) of Korea Grant funded by the Korean Governments by the Korea government (MSIP) [2013R1A1A2011999], [NRF-2013R1A2A2A01067194], and [2011-0030130]. The funders had no role in study design, data collection and analysis, decision to publish, or preparation of the manuscript.

## Author contributions

E-J Jin designed and performed the experiments, analyzed the data, and wrote the manuscript. J Song and D Kim performed the experiments and analyzed the data. C-H Chun provided crucial reagents and analyzed the data.

## Conflict of interest

All authors state that they have no conflict of interest.

## Abbreviations

OA: osteoarthritis
miR: microRNA
SHMT-2: serine hydroxymethyltransferase
MECP-2: methyl-CpG-binding protein 2
MTX: methotrexate
ADAMTS-5: A disintegrin and metalloproteinase with thrombospondin motifs 5
MMP: matrix metalloproteinase
5-mC: 5-methylcytosine
5-hmC: 5-hydroxymethylcytosine
TET: Tet methylcytosine dioxygenase
DHFR: dihydrofolate reductase
TYMS: thymidylate synthase
DNMT: DNA methyltransferase
UTR: untranslated region
