# Corrigendum

**DOI:** 10.1111/acel.13066

**Published:** 2019-12-05

Song, J., Kim, D., Chun, C. and Jin, E. (2015). miR‐370 and miR‐373 regulate the pathogenesis of osteoarthritis by modulating one‐carbon metabolism via SHMT‐2 and MECP‐2, respectively. *Aging Cell*, 14: 826–837. https://doi.org/10.1111/acel.12363

In the article, “miR‐370 and miR‐373 regulate the pathogenesis of osteoarthritis by modulating one‐carbon metabolism via SHMT‐2 and MECP‐2, respectively,” the authors would like to correct two sentence, few typos, and the label of Figure 2B, the graph of Figure 3B, the image of safranin O staining in Figure 4F of DMM/anti‐miR‐370 cartilage, the bottom label of the graph in Figure 6a, and the last two images of safranin O staining in Figure 6f of DMM/MECP2 cartilage as these were misplaced. 

The sentence is: (b) SiRNA‐mediated knockdown of SHMT‐2 was confirmed by immunoblotting (left upper panel).

It should read as: (b) SiRNA‐mediated knockdown of SHMT‐2 (siSHMT‐2) was confirmed by immunoblotting (left upper panel, *, siSHMT‐2 used for further studies).

The sentence is: Annexin V staining revealed that the induction of miR‐373 in OA chondrocytes sifnficanty reduced this apoptotic cell death to 2.26%, whereas inhibition of miR‐370 increased cell death up to 10% in normal chondrocytes.

It should read as: Annexin V staining revealed that the induction of miR‐373 in OA chondrocytes significantly reduced this apoptotic cell death to 2.26%, whereas inhibition of miR‐373 increased cell death up to 10% in normal chondrocytes. In the result for Figure 2B middle panel, "significantly increased" should read as "significantly decreased", in the result for Figure 4E, "reduced miR‐370 level and apoptosis" should read as "reduced miR‐370 and induced apoptosis, and in the legend of Figure 6F, miR‐370 should read as miR‐373. The correct version of the Figures 3B, 4F, 6A, and 6F are shown below:

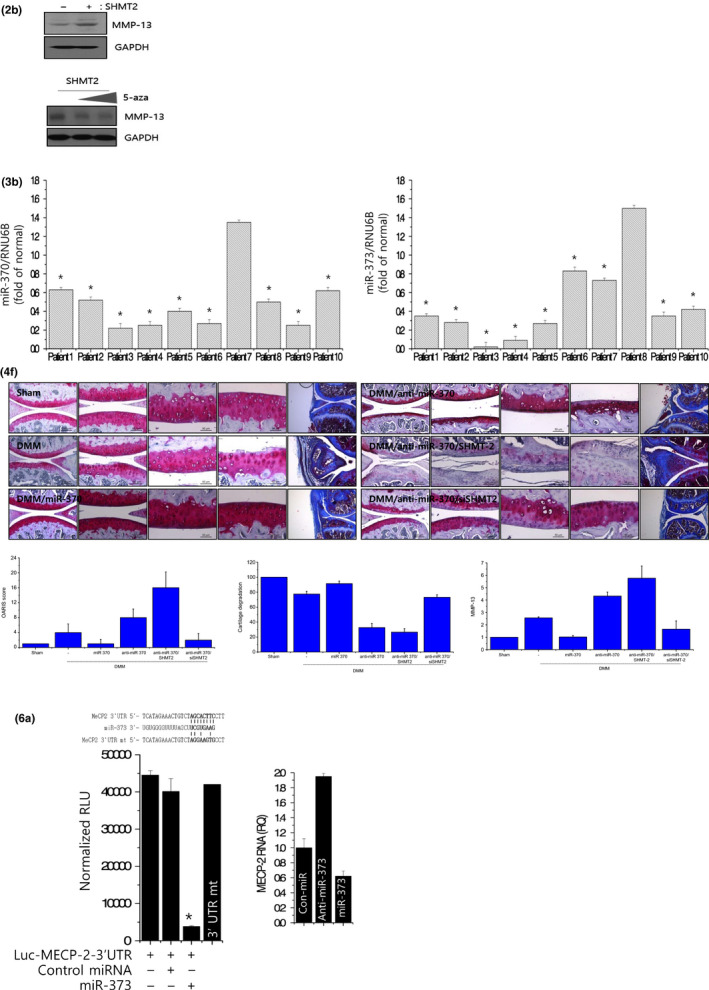

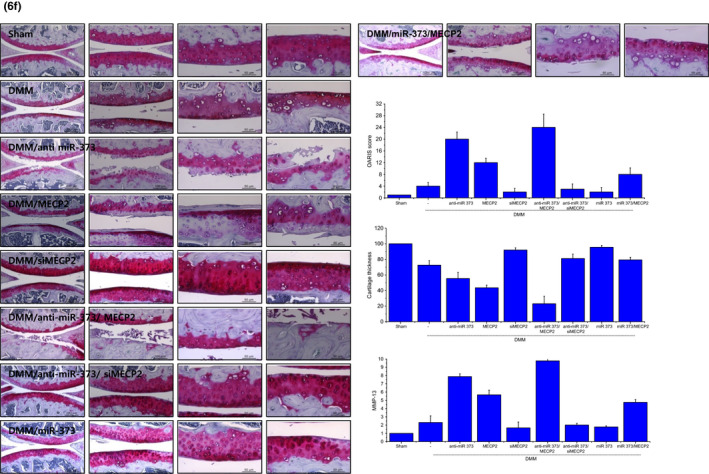

The authors would like to apologize for the inconvenience caused.

